# Enhancing Signal Output and Avoiding BOD/Toxicity Combined Shock Interference by Operating a Microbial Fuel Cell Sensor with an Optimized Background Concentration of Organic Matter

**DOI:** 10.3390/ijms17091392

**Published:** 2016-08-24

**Authors:** Yong Jiang, Peng Liang, Panpan Liu, Yanhong Bian, Bo Miao, Xueliang Sun, Helan Zhang, Xia Huang

**Affiliations:** State Key Joint Laboratory of Environment Simulation and Pollution Control School of Environment, Tsinghua University, Beijing 100084, China; jiangyong13@mails.tsinghua.edu.cn (Y.J.); liuxin8906@gmail.com (P.L.); byh13@mails.tsinghua.edu.cn (Y.B.); mb15@mails.tsinghua.edu.cn (B.M.); sun-xl14@mails.tsinghua.edu.cn (X.S.); hlzhang1126@gmail.com (H.Z.); xhuang@tsinghua.edu.cn (X.H.)

**Keywords:** biosensor, microbial fuel cell, toxicity, signal interference

## Abstract

In the monitoring of pollutants in an aquatic environment, it is important to preserve water quality safety. Among the available analysis methods, the microbial fuel cell (MFC) sensor has recently been used as a sustainable and on-line electrochemical microbial biosensor for biochemical oxygen demand (BOD) and toxicity, respectively. However, the effect of the background organic matter concentration on toxicity monitoring when using an MFC sensor is not clear and there is no effective strategy available to avoid the signal interference by the combined shock of BOD and toxicity. Thus, the signal interference by the combined shock of BOD and toxicity was systematically studied in this experiment. The background organic matter concentration was optimized in this study and it should be fixed at a high level of oversaturation for maximizing the signal output when the current change (Δ*I*) is selected to correlate with the concentration of a toxic agent. When the inhibition ratio (IR) is selected, on the other hand, it should be fixed as low as possible near the detection limit for maximizing the signal output. At least two MFC sensors operated with high and low organic matter concentrations and a response chart generated from pre-experiment data were both required to make qualitative distinctions of the four types of combined shock caused by a sudden change in BOD and toxicity.

## 1. Introduction

To preserve the water quality safety, the monitoring of pollutants (e.g., biochemical oxygen demand, BOD and toxicity) in an aquatic environment is important for mitigating the impact of a sudden contamination. The conventional physiochemical analysis methods, which can precisely measure the total concentration of target agents, are generally not suitable for on-line and on-site water monitoring because of the requirement of complex analysis protocols, skilled personnel, and technologically advanced large machinery. Generic biosensors are widely used for water monitoring, which can provide information on the bioavailable concentration and biological response to a wide range of chemical mixtures [1,2]. Early works have indicated that the microbial fuel cell (MFC) can be used as a sustainable and on-line electrochemical microbial biosensor for BOD and toxicity monitoring, respectively [3,4]. In the MFC sensor, electrogenic microorganisms inside the anode biofilm catalyze the oxidizing of organic matter, and the electrons released during the oxidizing process are captured by the anode through a particular extracellular electron transfer (EET) process. The captured electrons are further transferred through the external circuit, where the output signals were recorded, and finally reach the cathode where electron acceptors (e.g., oxygen and ferricyanide) are reduced [5,6]. The MFC can serve as a self-sustainable, low-cost, and user-friendly sensor for water monitoring without a driving power source or additional transducer, which is generally required in other types of biosensors to convert the original output signal to a quantitative electrical signal [7].

The activity of electrogenic microorganisms generally increases with the increase of the dissolved organic matter until the concentration reaches saturation, according to the Michaelis-Menten equation [4]. For toxicity monitoring, the MFC sensors are generally used in turn-off mode and the activity of the electrogenic microorganisms can be suppressed by the sudden leakage of toxic agents in water, leading to a decrease in the electric signal output [8,9]. Obviously, a certain concentration of organic matter in the anolyte is required to sustain the activity of the electrogenic microorganisms and thus maintain a certain degree of background signal output of an MFC sensor for toxicity monitoring. Generally, the biological toxicity of the target toxic agents is evaluated by correlating the concentration of toxic agents with the output electric signal, i.e., current change (Δ*I*) and inhibition ratio (IR). The Δ*I* is used because it is expedient to obtain the sensitivity, which is commonly selected as the target parameter when optimizing the sensor, by simply normalizing the Δ*I* to the toxic agent concentration. In contrast, IR is used when it is more suitable to evaluate the toxic effect of the target toxic agents and make a comparison between different studies, because IR presents the amplitude rather than the value of change in the electric signal [3,8]. However, it is still not clear with what kind of background organic matter concentration a maximization of the signal output can be achieved for toxicity monitoring. The different methods of signal resolution and the variation in the selection of indicators for biological toxicity in MFC toxicity sensor studies further increase the complexity between the background organic matter concentration and the maximization of the signal output.

In practice, the sudden change of BOD and toxicity could simultaneously occur when using the MFC sensor in field tests [10,11]. Many waste streams, such as animal farm effluence, rayon industry extracts and petrochemical factory pollutants, contain both organic matter and toxic agents [10]. The risk of a sudden change of BOD and toxicity further increases when there are several sewage outlets along the receiving water body. Based on the working principle of the MFC sensor, there is a decreased current output in response to the toxicity while there is an increased current output in response to BOD. The sudden change of BOD is thus the most likely to weaken or even mask the responses of the MFC sensor to toxicity. A recent study indicated that the availability of organic substrate to the anode biofilm affected the signal output from a combined shock of BOD and toxicity by challenging the MFC sensor with the combined shock of an acetate/Cr(VI) mixture [12]. However, the signal interference caused by the sudden change of BOD and toxicity when using the MFC sensor for water monitoring was not systematically studied. Moreover, there is no strategy available as of yet to avoid the signal interference in the combined shock of BOD and toxicity.

In this study, the background organic matter concentration was optimized for maximization of the signal output when using an MFC sensor for toxicity monitoring. Moreover, the signal interference caused by the sudden change of BOD and toxicity was systematically studied and a strategy was proposed to avoid it. By operating two MFC sensors with high and low background organic matter concentrations, respectively, the four types of combined shocks should be qualitatively distinguished after the comparison of a response chart.

## 2. Results

### 2.1. Organic Matter Detection and Typical Concentrations Selection

**Figure 1:**
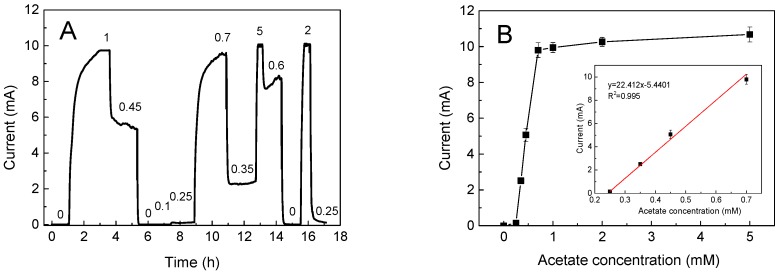
The use of the microbial fuel cell (MFC) sensor for organic matter detection: (**A**) the use of the MFC sensor for organic matter detection (0–5 mM) in a continuous flow-through mode; (**B**) the correlation of the output plateau current with the acetate concentration, which was used to select the typical background concentrations.

The MFC sensor was first used for organic matter detection in a continuous flow-through mode, as show in Figure 1A. The stepwise change in the acetate concentration (0–5 mM) in the anolyte led to the corresponding change in the current output. The correlation of the output plateau current with the acetate concentration was shown in Figure 1B. The lowest detection limit of the MFC sensor was 0.25 μM acetate, while the acetate concentrations (0.25–0.7 mM) and current output were highly correlated with *R*^2^ values >0.99. The response time significantly varied with a range from 20 min to 2 h depending on the acetate concentrations, which was in accordance with previous studies using an MFC which held a similar volume in the anode chamber [13,14]. Three acetate concentrations were selected for the following background organic matter optimizing test: 0.3 mM (near the detection limit), 0.5 mM (half-saturated maxing current), and 5 mM (oversaturation).

### 2.2. Background Organic Matter Concentration Affects the Toxicity Detection

**Figure 2:**
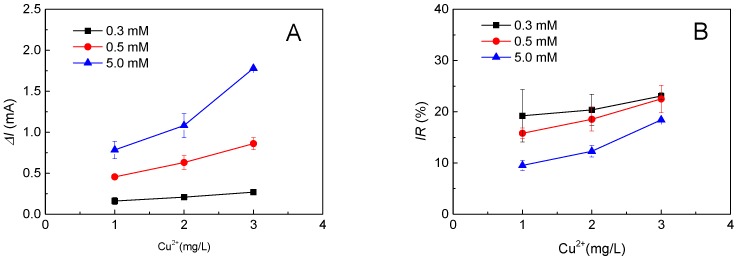
The background organic matter concentration affects the toxicity monitoring: (**A**) the effect of background organic matter concentration on the current change (Δ*I*) of the MFC sensor for toxicity monitoring; (**B**) the effect of background organic matter concentration on the inhibition ratio (IR) of the MFC sensor for toxicity monitoring.

The effect of the background organic matter concentration on the performance of the MFC sensor for toxicity monitoring was evaluated. As shown in Figure 2A, when Δ*I* is selected to correlate the concentration of a toxic agent, 1–3 mg/L of Cu(II), the background organic matter concentration should be fixed at a high level of oversaturation to maximize the signal output. However, as shown in Figure 2B, when IR is selected to correlate the concentration of a toxic agent, the background organic matter concentration should be fixed at a low level near the detection limit to minimize the signal output.

### 2.3. Signal Interference by Combined Shock of BOD and Toxicity

**Figure 3:**
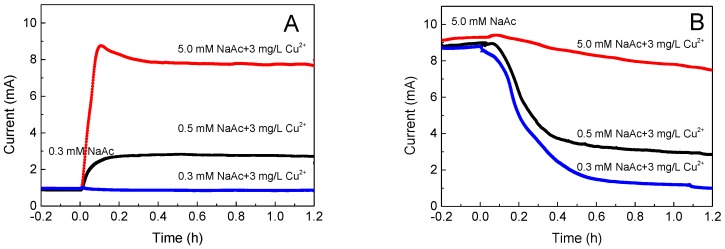
The signal interference of an MFC sensor by the combined shock of biochemical oxygen demand (BOD) and toxicity in a continuous flow-through mode: (**A**) the MFC sensor operated with background acetate of 0.3 mM; (**B**) the MFC sensor operated with background acetate of 5 mM.

In this study, this situation of a combined shock of BOD and toxicity was simulated by conducting the acetate (0.3, 0.5 and 5 mM)/Cu(II) (3 mg/L) combined shocks in a continuous flow-through mode to three MFC sensors. Specifically, when the background organic matter concentration was fixed at 0.3 mM, the current generation experienced a sharp increase in the combined shock of 0.5 mM acetate/3 mg/L Cu(II) and 5 mM acetate/3 mg/L Cu(II), while it showed a slight drop in the combined shock of 0.3 mM acetate/3 mg/L Cu(II) (Figure 3A). When the background organic matter concentration was fixed at 5 mM, the current generations all experienced a sharp decrease in the combined shock of 0.3 mM acetate/3 mg/L Cu(II), 0.5 mM acetate/3 mg/L Cu(II), and 5 mM acetate/3 mg/L Cu(II) (Figure 3B). These results clearly depict the signal interference by the combined shock of BOD and toxicity, and the trend and the amplitude of the output signal was determined by the concentration of the organic matter/toxic agents, as well as the background organic matter concentration.

**Figure 4:**
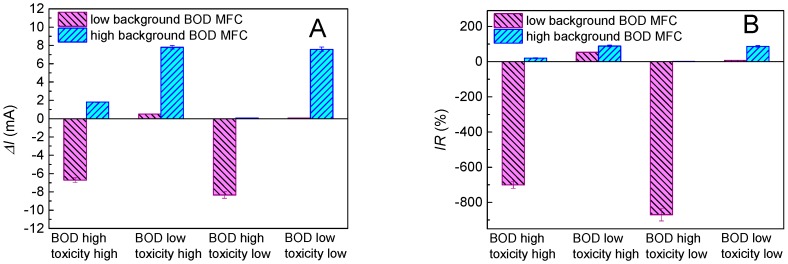
Response chart generated from MFC sensors run with high and low background concentrations of organic matter, respectively. Four types of combined shock caused by BOD and toxicity can be qualitatively distinguished using the response chart based on the Δ*I* (**A**) and IR (**B**).

Based on the above results, a response chart was generated and proposed as a strategy to avoid the signal interference in the combined shock of BOD and toxicity (Figure 4). At least two MFC sensors were required and operated with high and low organic matter concentrations, preferably with concentrations near the detection limit and oversaturation. In particular, the toxic effect of a target toxic agent in the MFC sensor was found not affected by the background organic matter concentration, but significantly affected by the organic matter concentration of the combined shock (Table S1). Thus, the increase in the organic matter concentration of the combined shock brought in a larger number of Δ*I*. By operating two MFC sensors with high and low background organic matter concentrations, respectively, different types of combined shock caused by BOD and toxicity should lead to different responses of MFC sensors, represented as the variation in Δ*I* and IR, and can be further qualitatively distinguished after the comparison of the response chart in Figure 4.

## 3. Discussion

Relatively larger deviations in the IR were observed with the low background organic matter concentration. This might be because the background organic matter concentration is near the minimum requirements for current generation. In this case, the current generation more easily fluctuated with respect to the aquatic environmental conditions. Generally, an increase in the background organic matter concentration led to a higher current generation, which provided a good deal of room for the drop in the signal, and thus a larger Δ*I* was observed. However, a higher current generation leads to a higher migration of ions rather than protons or hydroxyl ions to balance the charge in a neutral condition. The increased flux of the charged toxic cations, e.g., Cu(II), away the anode biofilm reduced the bioavailable toxic agents. In addition, the increased flux of organic acid anions, e.g., acetate, towards the anode biofilm increased the bioavailable organic matter [15,16]. For instance, a previous study proved that as the current density increased from 0.1 to 1.4 A/m^2^, the maximum concentration of the positively charged nickel ion in the bulk of the anodic compartment in an MFC sensor significantly decreased from 80 to 60 mg/L [15]. When the MFC sensor operated with a high background concentration of organic matter, the reduced bioavailable toxic agent concentration and increased bioavailable organic matter concentration, all caused by higher current generation, can synergistically lead to the decrease of the IR.

Typically, there are four types of combined shocks caused by BOD and toxicity: the BOD high/toxicity high, the BOD low/toxicity high, the BOD high/toxicity low, and the BOD low/toxicity low. These four types of combined shock can cause different signal outputs, which were recorded to calculate the Δ*I* and IR. As shown in Figure 4, the type BOD high/toxicity high and the BOD high/toxicity low can be easily distinguished because the Δ*I* and IR can be represented as a negative number for the MFC sensor when operated with a low background concentration of organic matter. Among these two types of shock, BOD high/toxicity high can be further distinguished because the Δ*I* and IR were significantly larger than those of BOD high/toxicity low when the MFC sensor operated with a high background concentration of organic matter. The remaining two types of combined shock, BOD low/toxicity high and BOD low/toxicity low, could not be easily distinguished from the response chart based on Δ*I* (Figure 4A). However, in the response chart based on IR (Figure 4B), it clearly showed that the combined shock of BOD low/toxicity high brought a much higher IR for the MFC sensor that operated with a low background concentration of organic matter.

In this study, the model organic matter and the model toxic agent were selected as acetate and copper, respectively. However, other types of BOD and toxic agents are expected to present a similar reaction when challenging the MFC sensor with BOD/toxicity combined shocks, but with different dose-dependent responses. A similar response chart is also expected, but of course with different amplitude. In summary, the change types of BOD and toxic agents can only change the related optimal background organic matter concentration and the amplitude and the response chart, but have no effect on the effectiveness of the method proposed in this study.

The response of the MFC sensor to BOD and toxic agents depends on the species composition of the anode biofilm, and the long-term stability of the anode biofilm is essential for long-term operation in field water monitoring. The four typical types of BOD/toxicity combined shocks can be distinguished by running the MFC sensors with different background organic matter concentrations. However, the difference in operating conditions can potentially change the species composition and the ecological shifts of the biofilm. In regards to practical water monitoring, the time scale of toxicity shock is relatively short compared with its normal state. Thus, the selection of resistant species in the MFC sensor was limited in regards to long-term practical water monitoring. To assure the fidelity of the response in long-term operations, the MFC sensors operated in different organic matter concentrations should switch among each other.

In conclusion, by operating two MFC sensors with high and low background organic matter concentrations and comparing the signal output with the pre-made response chart, this study provides a simple and easy method to qualitatively distinguish the four typical types of combined shocks caused by BOD/toxicity.

## 4. Materials and Methods

### 4.1. Construction of the MFC Sensor

A schematic of the two chamber MFC sensor is shown in Figure S1. The cubic anode and cathode chamber with liquid volumes of 8.4 mL and 28 mL, respectively, were separated by the cation exchange membrane (CMI7000, Membranes International Inc., Glen Rock, NJ, USA). A piece of graphite felt (Sanye Carbon Co., Ltd., Beijing, China) with 3 cm in diameter and a thickness of 0.2 cm was used as the anode, while a carbon-fiber brush of 3 cm long and 3 cm in diameter was used as the cathode. In accordance with our previous study, the anolyte was forced to flow through the porous anode, to improve the sensitivity [17]. A saturated calomel electrode (SCE, 242 mV versus the standard hydrogen electrode; Leici Co., Ltd., Shanghai, China) as the reference electrode was inserted into the anode chamber near the anode. All potentials presented were referenced to the SCE.

### 4.2. Startup of the Microbial Fuel Cell

The two chamber MFC sensor was inoculated by a mixed culture with the effluent of an acetate-fed MFC in our laboratory [18]. The anolyte was prepared by dissolving 0.82 g sodium acetate, 0.125 g NH_4_Cl, 0.332 g NaH_2_PO_4_·2H_2_O, 1.032 g Na_2_HPO_4_·12H_2_O, 0.13 g KCl, 5 mL vitamins solution, and 12.5 mL trace minerals solution in 1 L deionized water. The catholyte contained 100 mM K_3_Fe(CN)_6_ in 50 mM PBS solution in deionized water. Both the anolyte and catholyte were recirculated at a fixed flow rate of 5 mL/min using a multichannel peristaltic pump. The anode potential was controlled at +0.1 V using an eight-channel potentiostat (CHI 1030B, Chenhua Instruments Co., Ltd., Shanghai, China). Our previously study shown that the electrochemical deposition of copper was inevitable when the anode potential was set below −0.07 V [17]. Thus a relatively high anode potential was fixed here to avoid the electrochemical reduction and electrodeposition of copper.

### 4.3. Biosensor Test

After the startup process, the MFC sensors were then fed with a throughput of a sample stream during the process of the biosensor test, while the flow rate was still fixed at 5 mL/min. The anolyte was modified with specific concentrations of sodium acetate (as stated in the Section 3) to test the MFC sensor for organic matter detection. The typical background concentrations of acetate were selected according to the calibration curve to maximize the signal output. For toxicity monitoring, the MFC sensor was challenged with anolyte containing (per liter) specific concentrations of NaAc and Cu(II) (note: Copper chloride, CuCl_2_) and 0.13 g NH_4_Cl. The copper was chosen here as one of the typical heavy metal ions in water contaminants, and it was commonly used as a model toxic agent in the studies of MFC sensors [19]. It should be noted that based on the relatively low concentrations of the acetate and the copper used in this study, the chemical precipitation of copper acetate (the solubility at 25 °C of 6.79 g/100 mL) was avoided. The hydraulic retention time (HRT) was calculated to be 1.68 min considering the volume of the anodic chamber was 8.4 mL. The each run was duplicated, and performed at room temperature of 25 ± 1 °C.

### 4.4. Analyses

The response time is defined as the time required to reach 95% of the new steady-state current generation after the change of a certain organic matter concentration [20]. The detection limit is defined as the concentration of target compound established by using a certain signal-to-noise ratio 3:1 [21]. The Δ*I* is defined as the value of current drop after the exposing of toxic agents or a combined shock.

(1) $$\Delta I=I_{nor}-I_{tox}$$

where *I_nor_* (mA) is the current generated before the exposing of toxic agents, *I_tox_* (mA) is the current output directly following the introduction of heavy metal.

The IR is defined as the percentage of current drop normalized to the current before the exposing of toxic agents, and calculated by the following equation [19]:

(2) $$\text{IR}(\%)=100\times (I_{nor}-I_{tox})/I_{nor}$$

In particular, the combined shock of BOD and toxicity was evaluated in this study and the value of both Δ*I* and IR can be represented as positive and negative numbers.

## 5. Conclusions

In this study, the background organic matter concentration was optimized for a maximization of the signal output when using an MFC sensor for toxicity monitoring. The background organic matter concentration should be fixed as high at oversaturation to maximize the signal output when Δ*I* is selected to correlate with the concentration of toxic agent, while it should be fixed as low at near the detection limit to maximize the signal output when IR is selected. Two MFC sensors operated with different background organic matter concentrations and a response chart generated from previous experimental data were required to avoid the signal interference in the combined shock of BOD and toxicity. Future work will focus on the long-term stability of the MFC sensors, and the detection algorithms using signals from mass data of the combined shock of BOD and toxicity for quantitative analysis.

## Supplementary Materials

Supplementary materials can be found at www.mdpi.com/1422-0067/17/9/1392/s1.
