# Supplementary material for: Enhancing Signal Output and Avoiding BOD/Toxicity Combined Shock Interference by Operating a Microbial Fuel Cell Sensor with an Optimized Background Concentration of Organic Matter
Source: Int J Mol Sci. 2016 Aug 24;17(9):1392. doi: 10.3390/ijms17091392 (PMC5037672; doi:10.3390/ijms17091392)
Supplement: Supplementary file 1 [file ijms-17-01392-s001.pdf]

Supplementary Materials: Enhancing Signal Output and Avoiding BOD/Toxicity Combined Shock Interference by Operating Microbial Fuel Cell Sensor with Optimized Background Concentration of Organic Matter

Yong Jiang, Peng Liang, Panpan Liu, Yanhong Bian, Bo Miao, Xueliang Sun, Helan Zhang and Xia Huang

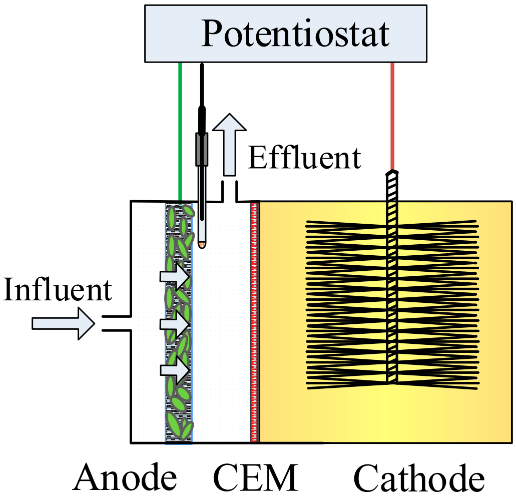

Figure S1. Schematic of the two chamber MFC sensor for toxicity monitoring.

Table S1. The effect of organic matter on the signal (Δ*I*) of 3 mg/L Cu(II) shock.

Organic Matter Concentration [a]	Δ*I* (mA)	
	**0.3 mM in Backgroud**	**5 mM in Backgroud**
5 mM in shock	1.626	1.809
0.5 mM in shock	1.191	1.061
0.3 mM in shock	0.430	0.229

[a] the organic matter here was a certain concentration of acetate.
